# The Low Down on e-Science and Grids for Biology

**DOI:** 10.1002/cfg.115

**Published:** 2001-12

**Authors:** Carole Goble

**Affiliations:** Department of Computer Science University of Manchester Oxford Road Manchester M13 9PL UK

## Abstract

The Grid is touted as a next generation Internet/Web, designed primarily to support e-Science. I hope to shed some light on what the Grid is, its purpose, and its potential impact on scientific practice in biology. The key message is that biologists are already primarily working in a manner that the Grid is intended to support. However, to ensure that the Grid’s good intentions are appropriate and fulfilled in practice, biologists must become engaged in the process of its development.


**Comparative and Functional Genomics**
*Comp Funct Genom* 2001; **2**: 365–370.
**DOI:** 10.1002/cfg.115

**Review**

### The low down on e-science and grids for biology

Carole Goble*
*Department of Computer Science,University of Manchester, Oxford Road, Manchester, M13 9PL, UK*

**Correspondence to:
Department of Computer Science
University of Manchester, Oxford
Road, Manchester, M13 9PL, UK.
E-mail: carole@cs.man.ac.uk*

Received: 10 October 2001
Accepted: 12 October 2001
Published online:
13 November 2001

### Abstract

**The Grid is touted as a next generation Internet/Web, designed primarily to support e-Science. I hope to shed some light on what the Grid is, its purpose, and its potential impact on scientific practice in biology. The key message is that biologists are already primarily working in a manner that the Grid is intended to support. However, to ensure that the Grid's good intentions are appropriate and fulfilled in practice, biologists must become engaged in the process of its development. Copyright © 2001 John Wiley & Sons, Ltd.**

**Keywords: Grid; e-Science; bioinformatics; *in silico* experiments; workflow; information management; analysis**

### Introduction

We are familiar with the idea of e-Commerce: the electronic trading between consumers and suppliers. E-Commerce reflects the globalisation of business and the way that commerce is changing. Similarly the way that science is done in biology is changing. **e-Science** is the use of electronic resources – instruments, sensors, databases, computational methods, computers – by scientists working collaboratively in large distributed project teams in order to solve scientific problems. Large-scale science, as illustrated by the Human Genome Project, will increasingly be carried out through distributed global collaborations, enabled by the Internet, which will require access to very large data collections, very large scale computing resources and high performance visualisation. In practice, biology has already moved to large interdisciplinary teams distributed throughout the world working together on specific problems. Post-genomics and high throughput experimentation is promising to overwhelm the community with an avalanche of data that needs to be organised and harnessed. The data is often complex, generated through different media, variable in quality, stored in many places, difficult to analyse, often changing and mostly comprised of incomplete data sets. Analysis methods to handle the different types of data are constantly and rapidly evolving. The questions we ask of the data, and the computational analyses to ask them, are more complicated: multiple species rather than single species; whole genome rather than single gene; whole metabolic lifecycle rather than single biological process. The computational power needed to model metabolic pathways or cells will be huge. Consequently, the traditional scientific experimental methods are supplemented with '*in silico* experiments', for example, the prediction of genes and the metabolic pathways they encode from the genomic DNA of an organism. Experiments *in silico* complement experiments *in vitro* by generating hypotheses for lab-based confirmation.

In the early 1990s web technology was rapidly taken on board by the biological community as a way of disseminating data and analysis methods that were readily accessible to the wider biology community. The Web enabled individual scientists to answer simple 'low volume' questions over large but relatively simple data sets without needing a profound knowledge of computer science. The sharing of data repositories and tool libraries became straightforward. Widespread collaboration was possible even if it was just by publishing a simple web page. However, standard web technology is now straining to meet the needs of biologists. The

Copyright © 2001 John Wiley & Sons, Ltd.

next step is a much more powerful infrastructure to generally support further growth of e-Science – the Grid. The Grid should enable collaborative groups of scientists to ask complex questions over complex data sets without a profound knowledge of computer science.

In October 2000, the UK government announced a £120 million programme to develop and deploy Grid technology to support the challenges of e-Science. The European Union announced a Grid programme. In the USA, where the term 'The Grid' was first coined, serious money has already been invested. IBM and Sun Microsystems have announced significant investment in the Grid. But just what is the Grid? And what does it mean for a biologist or a bioinformatician serving the biology community?

### What is the Grid?

'The Grid' is the name given to a proposed distributed computing infrastructure for advanced science and engineering. The name comes from an analogy with an electricity power grid – computing and data resources will be delivered over the Internet seamlessly, transparently and dynamically as and when needed. An e-Scientist should be able to plug into the e-Science computing infrastructure just like plugging into a power grid. Its origins lie in the requirements of high-energy physics, whose experiments generate petabytes of data in a few seconds, and whose simulations took months of computational processing. Thus, at the heart of the Grid lie high-speed networked communications, dynamic machine processor sharing and vast data handling. However, the Grid is now no longer just a synonym for networked high performance computing. It is a bigger vision of '¥flexible, secure, coordinated resource-sharing among dynamic collections of individuals, institutions, and resources – what we refer to as *virtual organisations*' [2] Resources in this context includes computational systems and data storage and specialised experimental facilities. Now the Grid is seen more as a platform to support coordinated resource sharing and problem solving on a global scale for data-intensive and compute-intensive applications.

The major differences between the Grid and the Web are in the increased computing power available, the increased volume of data that can be handled and the speed with which data can be transferred between nodes on the Grid. The Grid will also provide vast capacity to store and retrieve data from a variety of sources and will allow the presentation of data obtained in the same format, regardless of its source. The main thing is that for the Grid to work it must work seamlessly, and transparently, supporting the scientist but not supplanting them – you won't care where your calculation is done or where data is actually held, it will just happen. The success of the Grid will be when a bioinformatician, like a database curator, finds it easier to use than not, and a biologist only knows it's there when it breaks.

### The vision: a Grid-enabled scenario

Let's use a scenario to present the potential of a system that uses the Grid. Robert is a biologist in a team examining yeast gene expression. Before conducting a microarray experiment he has checked whether any other similar experiment has taken place and if the data was already available. The system recommends a set of parameters for the machine. A sample is logged into a database and labelled. The microarray machine, recognising Robert from the log, sets parameters to those he has used on previous runs. The parameters are recorded with the output results, which are stored in his personal database alongside the image results.

The results are immediately accessible by Robert from his office where he analyses them with a number of specialist statistical computations and a complex interactive time-series visualisation, both of which dynamically exploit a number of available computational resources to get better performance. The visualisation is examined collaboratively with a colleague on a remote site. Online personal notes are attached to the results by both scientists. Several products with up regulated expression look interesting. A search using the SRS database portal identifies this gene as encoding a transcription factor. Papers, in free text, quoted to the database entries and extracted online from the Medline digital library reveal that, in certain circumstances, it could control genes related to the yeast gene of interest. The system recommends other scientists who have published work or experiments that are related.

The system inspects Robert's lab's various transcriptome databases, and discovers genes that were co-regulated with the original gene also share a

Copyright © 2001 John Wiley & Sons, Ltd. 

target site. This information is added to a yeast database with a link to the workflow of database interrogations and analysis tools that lead to the discovery, including versions of databases, parameter settings, versions of the algorithms and the lab that made the discovery.

Other scientists with appropriate access rights to this database who have run an analysis that included the gene in the last month are automatically notified with this new information. Another scientist incorporates the results into a simulation of a metabolic pathway they are running, using a problem-solving environment. The simulation is monitored by various colleagues around the world, who record both private and public observations. The simulation and its results are added to a public database, and trigger new simulations automatically.

This scenario illustrates six major characteristics, and challenges, of the proposed Grid:

(i) **An open platform to facilitate interoperability**: the Grid plans to be a universal platform bridging heterogeneous systems. The Grid connects all the players in a scientific endeavour: the instruments and sensors; the databases and documents; the machines and networks and the people (e.g. via video). This platform must be scalable, be able to evolve to be future proof and be fault-tolerant, robust, persistent and reliable. Metadata (data about the data) describes the environment, the services available and the ways they can be combined and exploited. Resources are advertised, brokered, monitored and removed.

(ii) **Large scale distributed information management**: the Grid should store and process the huge volumes and diversity of content efficiently. Content can be combined from multiple sources in unpredictable ways depending on the users' needs, and users should be able to discover, transparently access and process relevant content wherever it is located on the Grid. New methods are needed for archiving, mining, manipulating and sharing information derived from multiple sources. Think of Napster (**http://www.napster.com/**) or an enhanced SRS (**http://srs.ebi.ac.uk/**) [1].

(iii) **The explicit management of experimental process or 'workflows'**: The 'workflows' – how database searches and analysis tools flow together to generate a result – are as important and exchangeable as the results they generate. Recording, and sharing, workflows helps: improve experimental practice by avoiding unnecessary replication of *in silico* experiments (or *in vitro* experiments for that matter); assist in setting up of equipment or computational processes in appropriate ways; and ensure that conclusions are not drawn that are not fully justified by the techniques used.

(iv) **Coordinated distributed resource sharing**: computationally intensive data analysis and predictive modelling can take advantage of spare resources available on machines connected to the Grid. Resources are discovered, allocated and disbanded dynamically and transparently to the user. Think of the SETI@home project (**http://setiathome.ssl.berkeley.edu/**) [3].

(v) **Collaborative science**: users will form, maintain and disband communities of resources, use video conferencing and shared collaborative environments to jointly solve problems.

(vi) **Governance services**: a distributed environment on the scale of the Grid requires a number of core services built into its fabric to govern the whole scientific environment: ownership and watermarking (who owns the resource); provenance, quality, audit, versioning (where did the data come from and when); authentication, security and confidentiality (who can access the resource); change management and propagation (has the data/workflow I'm using changed), personalisation and configuration (my lab book is special to me) and so on.

There are many ways of thinking about the Grid; three of these are different perspectives that complement one another. Figure 1 shows perspectives one and two; Figure 2 shows perspective three.

(i) A configuration of resources: geographically (a UK Grid); for a particular community (a Grid for mouse); to solve a particular problem (a Grid for simulations of protein folding); local (a Grid within a pharmaceutical company); or organised into tiers (as in the CERN Physics Grid). These configurations are dynamic that are formed, used and disbanded as and when needed.

(ii) An infrastructure oriented technology grid (of instruments, machines, software and data) that serves an access grid of people according to the governance of their community.

Copyright © 2001 John Wiley & Sons, Ltd.

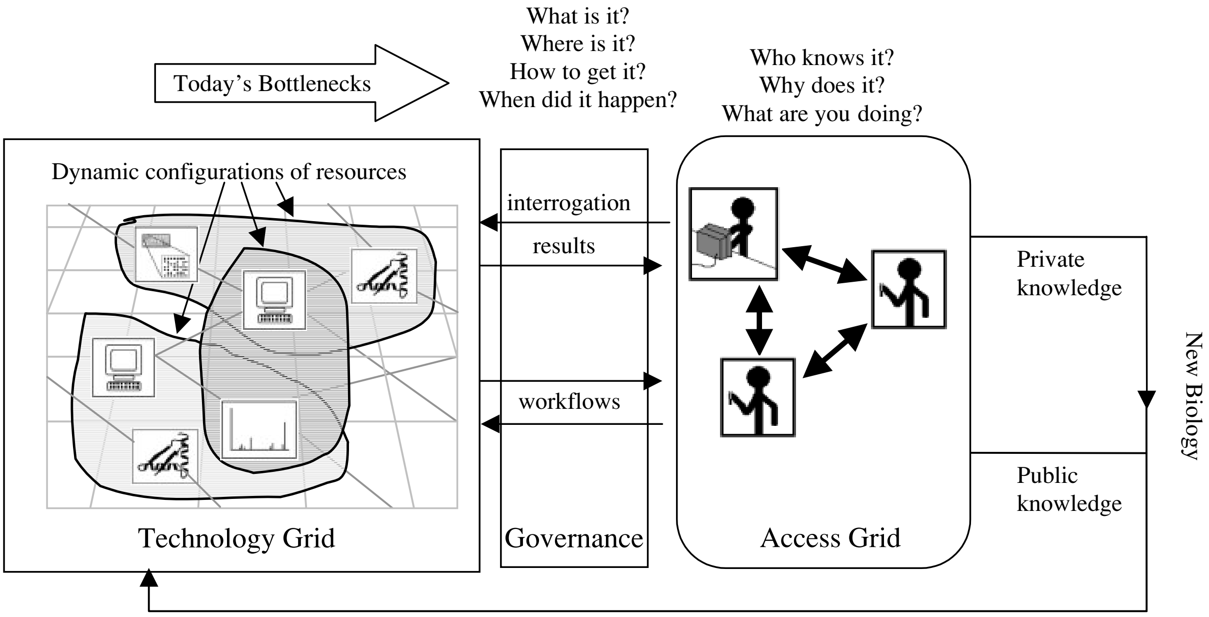

**Figure 1.** Two user views of the Grid. Configurations of shared resources serve configurations of co-operating scientists

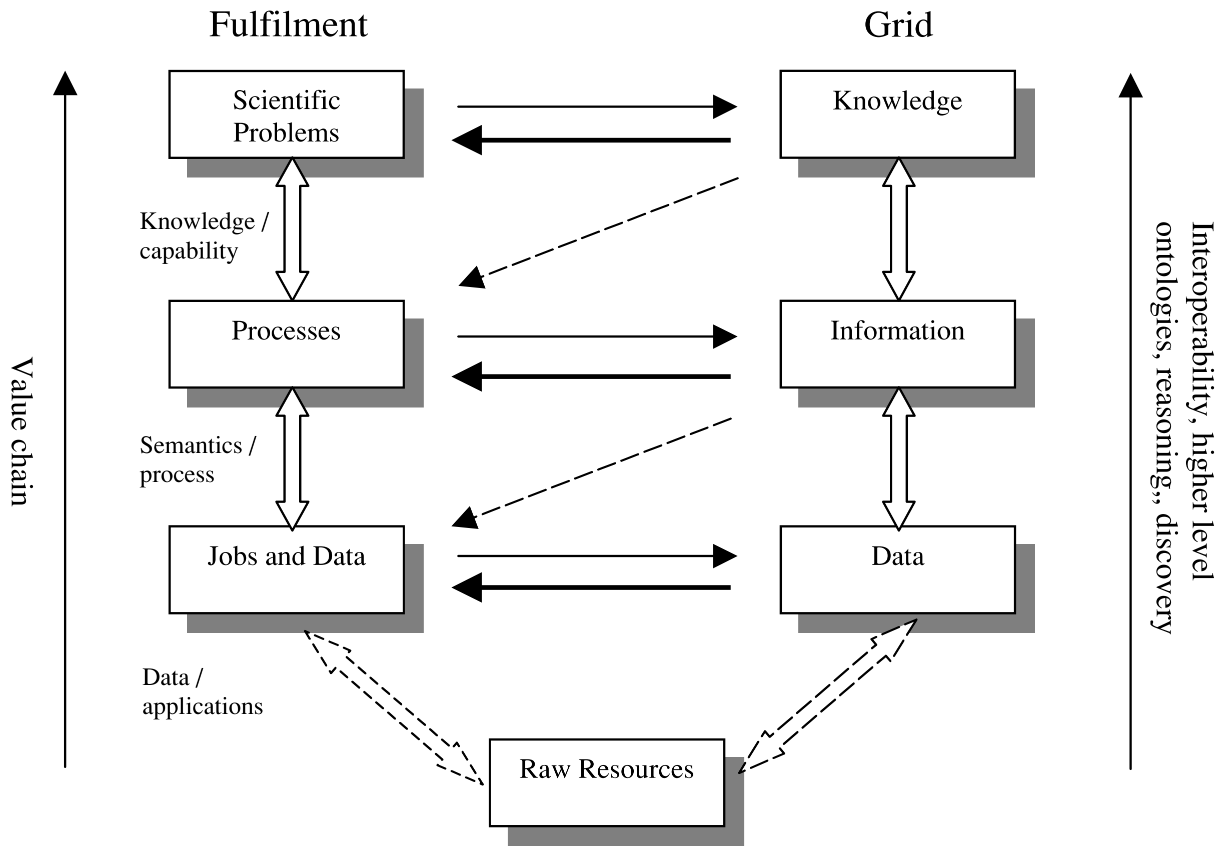

**Figure 2.** A technical view of the Grid. Reproduced by permission of the IT Innovation Centre, University of Southampton. **http://www.it-innovation.soton.ac.uk**

Copyright © 2001 John Wiley & Sons, Ltd. 

(iii) A stack of conceptual services: popularly presented as three layers, with built-in governance services at each layer.
(iv) **Data/computation services** respond to requests for computers and data stores in a secure and auditable fashion. This forms the fabric of the Grid for managing large volumes of data, fast networks and presenting diverse resources as a single meta-computer. So it deals with the way that computational resources are allocated, scheduled, and executed, and the way that data is shipped between processing resources.
(v) *Eg. Execute BLAST by balancing the computation load across three machines that are available at the right cost and I am authorised to use, and ship the results to query over a remote implementation of SRS.*
(vi) **Information services**, on top of the data/ computation layer, respond to requests for computational processes that may require several data sources and processing stages to achieve the desired result. The Grid bundles together the Web, and other well-known and current middleware technologies, incorporating them into one framework. This layer deals with the way that information is represented, stored, accessed, shared and maintained, and thus includes toolkits for visualisation, data management, instrumentation management and so on.
(vii) *Eg. execute BLAST against my protein, select the top five results from SWISS-PROT and cluster by their GO terms.*
(viii) **Knowledge services**, on top of the information services, respond to high-level questions and find the appropriate processes to deliver answers in the required form. This layer includes data mining, ontologies, portals and Problem Solving Environments (PSE) to support the way knowledge is acquired, used, retrieved, published and maintained to assist e-scientists to achieve their goals. An example of is a PSE is Cactus (**http:// www.cactuscode.org/**).
(ix) *Eg. What is the function of my protein?*

The key points of each viewpoint are that (i) configurations of resources are dynamic and flexible; (ii) problems are localised at each layer so they are simplified or become invisible to the next layer above, and (iii) the grid is as much about people as it is computers.

### Examples of biology Grid projects

This all sounds very exciting, but just as the Web started as a place where only a few enthusiasts would go, so the Grid is in its early development. Right now there is only really one working version of the Grid – the NASA PowerGrid (**http://www. ipg.nasa.gov/**). These are early days, and expectations should not be set too high. There are many technical and basic research challenges to overcome before the vision outlined in the scenario is the routine reality that the Web and the Internet are today. However, a number of pilots have already been started, or are about to start, in biology. These include:

- The EU funded **Data Grid** project developing the data/computational grid layer has a demonstrator in parasitology (**http://www.eu-datagrid.org**);
- The EU funded **Bio GRID**, part of the EuroGrid project, will develop an access portal for biomolecular modeling resources. Bio-GRID will develop interfaces to enable chemists and biologists to be able to submit work to High Performance Computing facilities (**http://www. eurogrid. org/wp1.html**);
- A prototype **BioSciences Grid**, funded by the UK BBSRC and the Wellcome Trust, links five molecular simulation groups in the UK;
- **BioOpera** is an extensible process support management system for virtual biology laboratories, which concentrates on managing complex compute-intensive computations (**http:// www.inf.ethz. ch/personal/bausch/bioopera/main.html**);
- **myGrid**, funded by the UK EPSRC e-Science programme, aims to be an e-Scientist workbench for data-intensive bioinformatics with an emphasis on data integration, workflow, personalisation and provenance (**http://www.mygrid.org.uk**).

### A call to arms

E-Science and the Grid are being driven by the need to solve real problems in science, and that includes some real and pressing problems in biology. It is not a public works scheme for computer scientists – it is reflecting best current practice, as demonstrated by the Human Genome project, rather than imposing an alien structure. For biologists, collaborative problem solving environments with built in support

Copyright © 2001 John Wiley & Sons, Ltd. 

mechanisms such as provenance, security, and confidentiality, become easier to build. For bioinformaticians, it means a platform that they can use to benefit their tools, at the cost of adding their resources to the Grid. That primarily means three things: (i) making their resources amenable to machine processing, not just 'point-and-click' navigation through a web browser; (ii) offering better descriptions of what their service does and how it does it; and (iii) possibly extending their services to take advantage of new features. Without at least (i) and (ii) the Grid won't work.

The Web was originally developed at CERN as a scheme for enabling physicists to exchange ideas. It worked because the physics community had a real problem and the computer scientists worked with them to solve their problem, not some other problem. The same applies to the Grid. The biology community must get fully engaged in the process of the Grid's development to make sure that the Grid is biology driven. So far, the physicists have taken the initiative on the Grid, but an infrastructure that supports the CERN Large Hadron Collider will not be appropriate for post-genomic comparative functional analysis. There is a real danger in this whole process: generating wonderful technological solutions, but for the wrong problem. Although the aim of the Grid is to build generic technologies, there comes a point where application dependencies may arise. For the Grid and e-Science to work for biologists they must become engaged in the process – not in developing the underlying infrastructure, but in guiding the computer scientists to ensure that the systems developed work in ways which are productive to biology. Because the Grid is still in its early stages, now is the time for it to be guided.

The USA and most European funding agencies are putting in place funding programmes for Grid. The Global Grid Forum (**http://www.gridforum.org**) is the arena for sharing developments on the Grid. Go there. See what is going on. Get involved with local Grid activities in your lab, department, university, discipline, country, and continent. If the Grid can be developed as a true collaboration between biology and computer science, then the Grid could provide as much of a revolution to the conduct of biology in this decade, as the web and bioinformatics were to it in the last.

### Acknowledgement

The author would like to thank Andy Brass and Robert Stevens for their insights, which helped shape this article. The author would also like to thank Mike Surridge and Matthew Addis of the IT Innovation Centre, University of Southampton for permission to reproduce Figure 2.

### References

1. Etzold T, Argos P. 1993. SRS– an indexing and retrieval tool for flat file data libraries. *Comput Appl Biosci* **9**: 49–57.
2. Foster I, Kesselman C, Tuecke S. 2001. The anatomy of the Grid: Enabling scalable virtual organizations. *Int J High Perform C* **15**(3): 200–222.
3. Sullivan WT, Werthimer D, Bowyer S, Cobb J, Gedye D, Anderson D. 1997. A new major SETI project based on project SERENDIP data and 100 000 personal computers. In *Astronomical and Biochemical Origins and the Search for Life in the Universe*, Cosmovici CB, Bowyer S, Werthimer D (eds). Editrice Compositori: Bologna, Italy; 729.

Copyright © 2001 John Wiley & Sons, Ltd. 
